# Supplementary material for: Congenital transmission of Chagas disease by vector circulation zone in Bolivia
Source: PLoS Negl Trop Dis. 2025 Oct 3;19(10):e0013591. doi: 10.1371/journal.pntd.0013591 (PMC12510653; doi:10.1371/journal.pntd.0013591)
Supplement: S1 Table — (DOCX) [file pntd.0013591.s001.docx]

S1 Table. Maternal characteristics by *T. cruzi* transmission status. SD: standard deviation

|  | Overall  (n = 238) | Transmitted | | P-value |
| --- | --- | --- | --- | --- |
|  |  | ***Yes***  ***(n = 19)*** | ***No***  ***(n = 219)*** |  |
| Demographics |  |  |  |  |
| Age, mean (SD) | 28.7 ± 6.8 | 28.8 ± 7.0 | 28.7 ± 6.8 | 0.94 |
| Education level |  |  |  | 0.90 |
| Incomplete high school or less | 147 (61.8%) | 12 (63.2%) | 135 (61.6%) |  |
| High school or more | 91 (38.2%) | 7 (36.8%) | 84 (38.4%) |  |
| Occupation |  |  |  | 0.84 |
| Homemaker | 170 (71.4%) | 14 (73.7%) | 156 (71.2%) |  |
| Manual labor | 22 (9.2%) | 1 (5.3%) | 21 (9.6%) |  |
| Student | 6 (2.5%) | 0 (0.0%) | 6 (2.7%) |  |
| Professional or office worker | 23 (9.7%) | 3 (15.8%) | 20 (9.1%) |  |
| Domestic services | 13 (5.5%) | 1 (5.3%) | 12 (5.5%) |  |
| Other | 4 (1.7%) | 0 (0.0%) | 4 (1.8%) |  |
| Family history of Chagas disease |  |  |  | 0.052 |
| Yes | 129 (54.2%) | 12 (63.2%) | 117 (53.4%) |  |
| No | 65 (27.3%) | 1 (5.3%) | 64 (29.2%) |  |
| Unknown | 44 (18.5%) | 6 (31.6%) | 38 (17.4%) |  |
| Recalls being bitten by triatomine bug | 106 (44.5%) | 8 (42.1%) | 98 (44.7%) | 0.59 |
| Hospital region |  |  |  | 0.61 |
| Santa Cruz | 158 (66.4%) | 14 (73.7%) | 144 (65.8%) |  |
| Cochabamba | 51 (21.4%) | 4 (21.1%) | 47 (21.5%) |  |
| Chuquisaca | 29 (12.2%) | 1 (5.3%) | 28 (12.8%) |  |
| Home characteristics |  |  |  |  |
| Vector circulation zone |  |  |  | 0.31 |
| Low | 99 (41.6%) | 10 (52.6%) | 89 (40.6%) |  |
| High | 139 (58.4%) | 9 (47.4%) | 130 (58.4%) |  |
| Triatomine bugs seen in home | 111 (46.6%) | 10 (52.6%) | 10 (4.6%) | 0.62 |
| Home construction |  |  |  |  |
| Mud walls | 51 (21.4%) | 4 (21.1%) | 47 (21.5%) | 0.92 |
| Brick and cement walls | 185 (77.7%) | 16 (84.2%) | 169 (77.2%) | 0.57 |
| Palm or reed ceiling | 27 (11.3%) | 3 (15.8%) | 24 (11.0%) | 0.54 |
| Home amenities |  |  |  |  |
| Electricity | 235 (98.7%) | 19 (100%) | 216 (98.6%) | 0.61 |
| Refrigerator | 176 (73.9%) | 16 (84.2%) | 160 (73.1%) | 0.29 |
| Television | 207 (87.0%) | 18 (94.7%) | 189 (86.3%) | 0.30 |
| Computer | 30 (12.6%) | 4 (21.1%) | 26 (11.9%) | 0.25 |
| Time lived in current residence, years | 17.2 ± 10.7 | 17.1 ± 11.2 | 17.2 ± 10.7 | 0.98 |
| Obstetric history |  |  |  |  |
| Number of total pregnancies | 3.0 ± 1.7 | 2.8 ± 1.3 | 3.0 ± 1.8 | 0.66 |
| Gravidity  Primigravida  Multigravida | 43 (18.1%)  195 (81.9%) | 2 (10.5%)  17 (89.5%) | 41 (18.7%)  178 (81.3%) | 0.37 |
| Birth type |  |  |  | 0.75 |
| Vaginal or assisted vaginal | 117 (49.2%) | 10 (52.6%) | 107 (48.9%) |  |
| Cesarean | 121 (50.8%) | 9 (47.4%) | 112 (51.1%) |  |
| Co-infections |  |  |  |  |
| RPR/VDRL | 4 (1.7%) | 0 (0.0%) | 4 (1.8%) | 0.55 |
| Toxoplasmosis | 35 (14.7%) | 2 (10.5%) | 33 (15.1%) | 0.30 |
