# Supplementary material for: Congenital transmission of Chagas disease by vector circulation zone in Bolivia
Source: PLoS Negl Trop Dis. 2025 Oct 3;19(10):e0013591. doi: 10.1371/journal.pntd.0013591 (PMC12510653; doi:10.1371/journal.pntd.0013591)
Supplement: S2 Table — (DOCX) [file pntd.0013591.s002.docx]

**S2 Table. Sensitivity analysis of maternal characteristics by *T. cruzi* vector circulation zone.** Limited to participants with at least one positive confirmatory test for Chagas disease (rapid test, IHA, or ELISA).

|  | Overall  (n = 222) | Vector circulation zone | | P-value |
| --- | --- | --- | --- | --- |
|  |  | ***High***  ***(n = 129)*** | ***Low***  ***(n=93)*** |  |
| Demographics |  |  |  |  |
| Age, mean (SD) | 29.1 ± 6.8 | 29.3 ± 6.9 | 28.8 ± 6.6 | 0.63 |
| Education level |  |  |  | 0.14 |
| Incomplete high school or less | 139 (62.6%) | 86 (66.7%) | 53 (57.0%) |  |
| High school or more | 83 (37.4%) | 43 (33.3%) | 40 (43.0%) |  |
| Occupation |  |  |  | 0.20 |
| Homemaker | 158 (71.2%) | 88 (68.2%) | 70 (75.3%) |  |
| Manual labor | 22 (9.9%) | 16 (12.4%) | 6 (6.5%) |  |
| Student | 4 (1.8%) | 2 (1.6%) | 2 (2.2%) |  |
| Professional or office worker | 21 (9.5%) | 10 (7.8%) | 11 (11.8%) |  |
| Domestic services | 13 (5.9%) | 11 (8.5%) | 2 (2.2%) |  |
| Other | 4 (1.8%) | 2 (1.6%) | 2 (2.2%) |  |
| Family history of Chagas disease |  |  |  | 0.27 |
| Yes | 122 (55.0%) | 66 (51.2%) | 56 (60.2%) |  |
| No | 59 (26.6%) | 35 (27.1%) | 24 (25.8%) |  |
| Unknown | 41 (18.5%) | 28 (21.7%) | 13 (14.0%) |  |
| Recalls being bitten by triatomine bug | 102 (45.9%) | 62 (48.1%) | 40 (43.0%) | 0.27 |
| Hospital region |  |  |  | 0.24 |
| Santa Cruz | 147 (66.2%) | 80 (62.0%) | 67 (72.0%) |  |
| Cochabamba | 50 (22.5%) | 34 (26.4%) | 16 (17.2%) |  |
| Chuquisaca | 25 (11.3%) | 15 (11.6%) | 10 (10.8%) |  |
| Home characteristics |  |  |  |  |
| Triatomine bugs seen in home | 109 (49.1%) | 76 (58.9%) | 33 (35.5%) | **<0.001** |
| Home construction |  |  |  |  |
| Mud walls | 48 (21.6%) | 40 (31.0%) | 8 (8.6%) | **<0.001** |
| Brick and cement walls | 176 (79.3%) | 93 (72.1%) | 83 (89.2%) | **0.002** |
| Palm or reed ceiling | 25 (11.3%) | 23 (17.8%) | 2 (2.2%) | **<0.001** |
| Home amenities |  |  |  |  |
| Electricity | 219 (98.6%) | 127 (98.4%) | 92 (98.9%) | 0.76 |
| Refrigerator | 165 (74.3%) | 90 (69.8%) | 75 (80.6%) | 0.067 |
| Television | 193 (86.9%) | 108 (83.7%) | 85 (91.4%) | 0.094 |
| Computer | 28 (12.6%) | 16 (12.4%) | 12 (12.9%) | 0.89 |
| Time lived in current residence, years | 17.5 ± 10.8 | 16.9 ± 10.0 | 18.3 ± 11.7 | 0.37 |
| Obstetric history |  |  |  |  |
| Transmission of *T. cruzi* to infant | 19 (8.6%) | 9 (7.0%) | 10 (10.1%) | 0.32 |
| Number of total pregnancies | 3.1 ± 1.8 | 3.0 ± 1.8 | 3.1 ± 1.7 | 0.70 |
| Gravidity  Primigravida  Multigravida | 37 (16.7%)  185 (83.3%) | 24 (18.6%)  105 (81.4%) | 13 (14.0%)  80 (86.0%) | 0.36 |
| Birth type |  |  |  | 0.95 |
| Vaginal or assisted vaginal | 108 (48.6%) | 63 (48.8%) | 45 (48.4%) |  |
| Cesarean | 114 (51.4%) | 66 (51.2%) | 48 (51.6%) |  |
| Co-infections |  |  |  |  |
| RPR/VDRL | 4 (1.8%) | 3 (2.3%) | 1 (1.1%) | 0.46 |
| Toxoplasmosis | 33 (14.9%) | 15 (11.6%) | 18 (19.4%) | 0.11 |
