# Supplementary material for: Congenital transmission of Chagas disease by vector circulation zone in Bolivia
Source: PLoS Negl Trop Dis. 2025 Oct 3;19(10):e0013591. doi: 10.1371/journal.pntd.0013591 (PMC12510653; doi:10.1371/journal.pntd.0013591)
Supplement: S3 Table — (DOCX) [file pntd.0013591.s003.docx]

S3 Table. Sensitivity analysis of maternal characteristics by hospital region. Limited to participants with at least one positive confirmatory test for Chagas disease (rapid test, IHA, or ELISA).

|  | Overall  (n = 222) | Santa Cruz  (n=147) | Cochabamba  (n=50) | Chuquisaca  (n=25) | P-value |
| --- | --- | --- | --- | --- | --- |
| Demographics |  |  |  |  |  |
| Age, mean (SD) | 29.1 ± 6.8 | 29.0 ± 7.0 | 30.4 ± 6.2 | 26.9 ± 6.3 | 0.58 |
| Education level |  |  |  |  | 0.47 |
| Incomplete high school or less | 139 (62.6%) | 89 (60.5%) | 35 (70.0%) | 15 (60.0%) |  |
| High school or more | 83 (37.4%) | 58 (39.5%) | 15 (30.0%) | 10 (40.0%) |  |
| Occupation |  |  |  |  | 0.63 |
| Homemaker | 158 (71.2%) | 101 (68.7%) | 37 (74.0%) | 20 (80.0%) |  |
| Manual labor | 22 (9.9%) | 14 (9.5%) | 7 (14.0%) | 1 (4.0%) |  |
| Student | 4 (1.8%) | 2 (1.4%) | 1 (2.0%) | 1 (4.0%) |  |
| Professional or office worker | 21 (9.5%) | 16 (10.9%) | 4 (8.0%) | 1 (4.0%) |  |
| Domestic services | 13 (5.9%) | 10 (6.8%) | 1 (2.0%) | 2 (8.0%) |  |
| Other | 4 (1.8%) | 4 (2.7%) | 0 (0.0%) | 0 (0.0%) |  |
| Family history of Chagas disease |  |  |  |  | **0.026** |
| Yes | 122 (55.0%) | 84 (57.1%) | 21 (42.0%) | 17 (68.0%) |  |
| No | 59 (26.6%) | 33 (22.4%) | 22 (44.0%) | 4 (16.0%) |  |
| Unknown | 41 (18.5%) | 30 (20.4%) | 7 (14.0%) | 4 (16.0%) |  |
| Recalls being bitten by triatomine bug | 102 (45.9%) | 59 (40.1%) | 30 (60.0%) | 13 (52.0%) | .11 |
| Home characteristics |  |  |  |  |  |
| Vector circulation zone |  |  |  |  | 0.24 |
| Low | 93 (41.9%) | 67 (45.6%) | 16 (32.0%) | 10 (40.0%) |  |
| High | 129 (58.1%) | 80 (54.4%) | 34 (68.0%) | 15 (60.0%) |  |
| Triatomine bugs seen in home | 109 (49.1%) | 65 (44.2%) | 31 (62.0%) | 13 (52.0%) | 0.12 |
| Home construction |  |  |  |  |  |
| Mud walls | 48 (21.6%) | 27 (18.4%) | 14 (28.0%) | 7 (28.0%) | 0.24 |
| Brick and cement walls | 176 (79.3%) | 116 (78.9%) | 38 (76.0%) | 22 (88.0%) | 0.45 |
| Palm or reed ceiling | 25 (11.3%) | 19 (12.9%) | 5 (10.0%) | 1 (4.0%) | 0.39 |
| Home amenities |  |  |  |  |  |
| Electricity | 219 (98.6%) | 146 (99.3%) | 49 (98.0%) | 24 (96.0%) | 0.37 |
| Refrigerator | 165 (74.3%) | 103 (70.1%) | 45 (90.0%) | 17 (68.0%) | **0.015** |
| Television | 193 (86.9%) | 125 (85.0%) | 47 (94.0%) | 21 (84.0%) | 0.24 |
| Computer | 28 (12.6%) | 12 (8.2%) | 11 (22.0%) | 5 (20.0%) | **0.021** |
| Time lived in current residence, years | 17.5 ± 10.8 | 19.5 ± 10.3 | 13.0 ± 9.6 | 8.1 ± 10.4 | 0.89 |
| Obstetric history |  |  |  |  |  |
| Total number of pregnancies | 3.1 ± 1.8 | 3.2 ± 1.7 | 2.9 ± 1.9 | 2.6 ± 1.6 | 0.45 |
| Gravidity  Primigravida  Multigravida | 37 (16.7%)  185 (83.3%) | 22 (15.0%)  125 (85.0%) | 11 (22.0%)  39 (78.0%) | 4 (16.0%)  21 (84.0%) | 0.51 |
| Birth type |  |  |  |  | **<0.001** |
| Vaginal or assisted delivery | 108 (48.6%) | 49 (33.3%) | 37 (74.0%) | 22 (88.0%) |  |
| Cesarean | 114 (51.4%) | 98 (66.7%) | 13 (26.0%) | 3 (12.0%) |  |
| Co-infections |  |  |  |  |  |
| RPR/VDRL | 4 (1.8%) | 2 (1.4%) | 2 (4.0%) | 0 (0.0%) | 0.43 |
| Toxoplasmosis | 33 (14.9%) | 22 (15.0%) | 2 (4.0%) | 9 (36.0%) | **<0.001** |
