# Supplementary material for: Congenital transmission of Chagas disease by vector circulation zone in Bolivia
Source: PLoS Negl Trop Dis. 2025 Oct 3;19(10):e0013591. doi: 10.1371/journal.pntd.0013591 (PMC12510653; doi:10.1371/journal.pntd.0013591)
Supplement: S4 Table — (DOCX) [file pntd.0013591.s004.docx]

**S4 Table. Sensitivity analysis of maternal characteristics by transmission status.** Limited to participants with at least one positive confirmatory test for Chagas disease (rapid test, IHA, or ELISA).

|  | Overall  (n = 222) | Transmitted | | P-value |
| --- | --- | --- | --- | --- |
|  |  | ***Yes***  ***(n = 19)*** | ***No***  ***(n = 203)*** |  |
| Demographics |  |  |  |  |
| Age, mean (SD) | 29.1 ± 6.8 | 28.8 ± 7.0 | 29.1 ± 6.8 | 0.84 |
| Education level |  |  |  | 0.96 |
| Incomplete high school or less | 139 (62.6%) | 12 (63.2%) | 127 (62.6%) |  |
| High school or more | 83 (37.4%) | 7 (3.7%) | 76 (37.4%) |  |
| Occupation |  |  |  | 0.83 |
| Homemaker | 158 (71.2%) | 14 (73.7%) | 144 (70.9%) |  |
| Manual labor | 22 (9.9%) | 1 (5.3%) | 21 (10.3%) |  |
| Student | 4 (1.8%) | 0 (0.0%) | 4 (2.0%) |  |
| Professional or office worker | 21 (9.5%) | 3 (15.8%) | 18 (8.9%) |  |
| Domestic services | 13 (5.9%) | 1 (5.3%) | 12 (5.9%) |  |
| Other | 4 (1.8%) | 0 (0.0%) | 4 (2.0%) |  |
| Family history of Chagas disease |  |  |  | 0.057 |
| Yes | 122 (55.0%) | 12 (63.2%) | 110 (54.2%) |  |
| No | 59 (26.6%) | 1 (5.3%) | 58 (28.6%) |  |
| Unknown | 41 (18.5%) | 6 (31.6%) | 35 (17.2%) |  |
| Recalls being bitten by triatomine bug | 102 (45.9%) | 8 (42.1%) | 94 (46.3%) | 0.62 |
| Hospital region |  |  |  | 0.65 |
| Santa Cruz | 147 (66.2%) | 14 (73.7%) | 133 (65.5%) |  |
| Cochabamba | 50 (22.5%) | 4 (21.1%) | 46 (22.7%) |  |
| Chuquisaca | 25 (11.3%) | 1 (5.3%) | 24 (11.8%) |  |
| Home characteristics |  |  |  |  |
| Vector circulation zone |  |  |  | 0.32 |
| Low | 93 (41.9%) | 10 (52.6%) | 83 (40.9%) |  |
| High | 129 (58.1%) | 9 (47.4%) | 120 (59.1%) |  |
| Triatomine bugs seen in home | 109 (49.1%) | 10 (52.6%) | 99 (48.8%) | 0.79 |
| Home construction |  |  |  |  |
| Mud walls | 48 (21.6%) | 4 (21.1%) | 44 (21.7%) | 0.91 |
| Brick and cement walls | 176 (79.3%) | 16 (84.2%) | 160 (78.8%) | 0.69 |
| Palm or reed ceiling | 25 (11.3%) | 3 (15.8%) | 22 (10.8%) | 0.53 |
| Home amenities |  |  |  |  |
| Electricity | 219 (98.6%) | 19 (100%) | 200 (98.5%) | 0.59 |
| Refrigerator | 165 (74.3%) | 16 (84.2%) | 149 (73.4%) | 0.30 |
| Television | 193 (86.9%) | 18 (94.7%) | 175 (86.2%) | 0.29 |
| Computer | 28 (12.6%) | 4 (21.1%) | 24 (11.8%) | 0.25 |
| Time lived in current residence, years | 17.5 ± 10.8 | 17.1 ± 11.2 | 17.5 ± 10.8 | 0.88 |
| Obstetric history |  |  |  |  |
| Number of total pregnancies | 3.1 ± 1.8 | 2.8 ± 1.3 | 3.1 ± 1.8 | 0.53 |
| Gravidity  Primigravida  Multigravida | 37 (16.7%)  185 (83.3%) | 2 (10.5%)  17 (89.5%) | 35 (17.2%)  168 (82.8%) | 0.45 |
| Birth type |  |  |  | 0.72 |
| Vaginal or assisted vaginal | 108 (48.6%) | 10 (52.6%) | 98 (48.3%) |  |
| Cesarean | 114 (51.4%) | 9 (47.4%) | 105 (51.7%) |  |
| Co-infections |  |  |  |  |
| RPR/VDRL | 4 (1.8%) | 0 (0.0%) | 4 (2.0%) | 0.54 |
| Toxoplasmosis | 33 (14.9%) | 2 (10.5%) | 31 (15.3%) | 0.28 |
