# Supplementary material for: Congenital transmission of Chagas disease by vector circulation zone in Bolivia
Source: PLoS Negl Trop Dis. 2025 Oct 3;19(10):e0013591. doi: 10.1371/journal.pntd.0013591 (PMC12510653; doi:10.1371/journal.pntd.0013591)
Supplement: S5 Table — (DOCX) [file pntd.0013591.s005.docx]

**S5 Table. Characterization of women with available parasite load.** Parasite load was available for a subset of 21 women.

|  | Age (years) | Vector circulation zone | Department | Infant hospitalization at birth | Transmitted *T. cruzi* infection | Parasite load (parasites/mL) |
| --- | --- | --- | --- | --- | --- | --- |
| Mother 1 | 23 | Low | Santa Cruz | Yes | Yes | 26.74 |
| Mother 2 | 29 | Low | Santa Cruz | No | No | 35.74 |
| Mother 3 | 21 | Low | Santa Cruz | No | No | 33.78 |
| Mother 4 | 25 | Low | Santa Cruz | No | Yes | 34.54 |
| Mother 5 | 30 | High | Santa Cruz | No | No | 33.47 |
| Mother 6 | 31 | Low | Santa Cruz | No | Yes | 33.04 |
| Mother 7 | 39 | High | Cochabamba | No | No | 33.61 |
| Mother 8 | 27 | High | Santa Cruz | No | No | 33.95 |
| Mother 9 | 24 | High | Santa Cruz | No | No | 28.41 |
| Mother 10 | 24 | Low | Cochabamba | No | Yes | 26.78 |
| Mother 11 | 38 | High | Santa Cruz | No | Yes | 26.98 |
| Mother 12 | 24 | High | Cochabamba | No | Yes | 24.64 |
| Mother 13 | 38 | Low | Santa Cruz | No | Yes | 25.78 |
| Mother 14 | 23 | High | Santa Cruz | Yes | Yes | 26.78 |
| Mother 15 | 15 | High | Santa Cruz | No | Yes | 25.89 |
| Mother 16 | 23 | High | Santa Cruz | No | Yes | 24.68 |
| Mother 17 | 31 | Low | Santa Cruz | No | Yes | 23.78 |
| Mother 18 | 20 | High | Chuquisaca | No | Yes | 26.96 |
| Mother 19 | 41 | Low | Cochabamba | No | Yes | 22.78 |
| Mother 20 | 29 | Low | Santa Cruz | No | Yes | 20.64 |
| Mother 21 | 34 | Low | Santa Cruz | No | Yes | 24.67 |
