# Supplementary material for: Congenital transmission of Chagas disease by vector circulation zone in Bolivia
Source: PLoS Negl Trop Dis. 2025 Oct 3;19(10):e0013591. doi: 10.1371/journal.pntd.0013591 (PMC12510653; doi:10.1371/journal.pntd.0013591)
Supplement: Attachment — Submitted filename: Response to Reviewers 1.6.25.docx [file pntd.0013591.s007.docx]

**Manuscript ID** PNTD-D-24-01292

Title: “Congenital Transmission of Chagas Disease by Vector Circulation Zone in Bolivia”

**Response to reviewer comments**

**Additional Editor Comments:**

Bolivia has the largest burden of Chagas disease worldwide, and congenital transmission accounts for a growing percentage of new cases. Thus, the authors initiated a prospective cohort study of women with Chagas disease and their infants at 11 hospitals in Bolivia. They sought to evaluate the epidemiology of congenital Chagas disease and characterize infant presentations in this endemic, high-burden setting. They also compared congenital transmission rates between communities with high and low vector (triatomine bug) circulation, a key characteristic tracked by the Bolivian National Chagas Program. There are several important differences in the characteristics of the women in the two zones. Still, there does not appear to be a significant difference in the risk of transmission of T. cruzi from mother-to-child in the two vector circulation zones.

Understanding the epidemiology and risk factors for congenital Chagas disease is crucial. Therefore, the attempt to tease the differences in congenital Chagas disease transmission stratified by vector circulation zone is fascinating. However, all of the reviewers point out that this manuscript suffers from flaws, mainly related to challenges with follow-up, a relatively small sample size, and results that are difficult to explain and understand. It is also essential to add a GIS map with three study regions, vectorial transmission zones (high and low), cases of mother with Chagas and infants with congenital transmission, and locations of participating medical facilities included for enrollment in Figure 11.

I agree with the comments raised by the reviewers and think it is crucial to satisfy most of comments raised by the reviewers to improve the quality and value of the manuscript.

**Reviewers' Comments:**

**Methods**

Reviewer #1: The objectives and study design are clearly described. The analysis is appropriate overall although it would be interesting to do an exploratory analysis to see if there is any association with parity and risk of mother-to-child transmission of T. cruzi. In particular, is transmission risk similar among primigravidae vs women in their second or third or more pregnancies? This could be potentially added to the sensitivity analysis since it only includes the mean number of pregnancies. And speaking of the sensitivity analysis, the purpose of this and how it was performed is not clearly described in the Methods.

- We added gravidity (primigravid vs. multigravida) to our analysis and updated Tables 1-2 and Supplemental Tables 1-4 accordingly. Transmission risk was not significantly different between primigravidae and multigravidae.
- We added clarifications in our methods to the purpose and technique of our sensitivity analysis: “Given the imperfect specificity of the OnSite Chagas Ab Combo Rapid Test (87.3% according to one recent study), each analysis was repeated in a sensitivity analysis limited to women who had at least one positive confirmatory test for Chagas disease through rapid test, IHA, or ELISA (15).”

  Two minor suggestions:

  1. For the OnSite Chagas Rapid Test, it would be helpful to provide the company name and city/country where it is produced.
- In our Methods, we added a complete description of the rapid test: “OnSite Chagas Ab Combo Rapid Test; CTK Biotech, United States.”

2. Treponema pallidum should be presented in italics.

- This change was made.

Reviewer #2: The definition of the target population is unclear.

- Our target population included all pregnant women who presented for delivery at the hospitals included in our study. As stated in the methods, “Participants were recruited from 11 participating hospitals within three Bolivian departments with *T. cruzi* endemicity: Santa Cruz, Cochabamba, and Chuquisaca (Figure 1).” Complete inclusion and exclusion criteria were described in the methods and included: “Pregnant women who presented to the hospital for delivery, had an identification card documenting prior diagnosis of Chagas disease, and provided informed consent were eligible to participate. …Women under the age of 18 were eligible to participate if their parents signed informed consent. Exclusion criteria included no prior diagnosis of Chagas disease or living in a municipality for less than one year. The latter criterion was included due to high levels of internal migration, particularly during harvest times, which could lead to incorrect classification of residence by vector circulation rate.”

Reviewer #3: Methods are clear and study design is adequate.
- Please include if any participants were lost to follow-up after enrollment. Both Mother and infants

- As we state in our Results, “1-month follow-up was completed by 224 infants (92.6%). ... 9-month follow-up was completed by 84 infants (34.7%). Our study did not involve follow-up testing for mothers.

- Please include your methods of determining infestation of triatomines and high circulation zones. This is discussed in the results section but how was this determined? In the introduction this is referenced from a manual cited from 2007. I think it is very important to include more details on this in the methods section as it is a major talking point in your discussion.

- Classification of vector circulation zones was provided directly by the directors of public health in each department (Servicio Departamental de Salud; SEDES). Study staff performed interviews with each health director in 2019 when the study was being designed, so vector circulation zones in our study are reflective of 2019 data. Health directors have access to internal data to the most recent epidemiological estimates from the National Chagas Program, which were not published for public use at the time. A more recent estimate of vector circulation zones in 2022 was later published in the National Action Plan (Figure 10). To make this clearer to readers, we added the following to the methods: “Vector circulation zones within each province were assigned directly by the director of each local health department (Servicio Departamental de Salud; SEDES) using internal data from the Bolivian National Chagas Program in 2019, when the study was designed.”

- Please discuss if the surveys and informed consents were in English, Spanish, Portuguese or other indigenous languages (Aymara, Quechua... which are spoken in these regions.

- We added the following sentence to our Methods: “Questionnaires were performed in person in Spanish (Supplement).”

- In the methods section I would discuss more details about the survey that was done and include a copy of the survey as a supplement. Was it done in person, native language, were they shown what a triatomine looked like? Which species?

- As above, we added the following sentence to our Methods: “Questionnaires were performed in person in Spanish (Supplement).” A copy of the questionnaire (in original Spanish) was added to the Supplement. Participants were not shown a picture of triatomine bugs as these are well known in these endemic regions. Furthermore, in the initial prenatal visit for pregnant women, doctors are expected to discuss the National Chagas Program and share a copy of its official fact sheet for pregnant women, which includes a picture of the triatomine bug.

**Results**

Reviewer #1: The analysis is consistent with the analysis plan as described in the Methods.

My only minor suggestion is to clarify in the description of parasite load that this refers to estimated load based on PCR.

If the data have been collected, it would be helpful for readers to understand the clinical manifestations of the newborns with congenital Chagas disease, especially those who were hospitalized, and to provide more information on their hospital course (duration of hospitalization, any complications, treatment with benznidazole).

Reviewer #2: The original analysis plan is not clear.

Reviewer #3: - I suggest adding a GIS map with three regions of the study, vectorial transmission zones (high and low), cases of mother with Chagas and cases with infants with congenital transmission. I would also suggest in the figure 11 participating medical facilities which were included for enrollment. A figure such as this really helps to tell the story which you are describing. Congenital transmission of T. cruzi is present at significant rate and vectorial transmission still exists despite tremendous integrated programs to tackle triatomine invasion into homes, this includes T. infestans as a major vector in this region of Bolivia.

- We thank the reviewer for these thoughtful suggestions and agree these figures provide an important visualization that adds value to our manuscript. We have added the following figures with GIS maps:
  - Figure 2: Distribution of participating hospitals and health centers in Bolivia.
  - Figure 3: Distribution of participating women with Chagas disease by province.
  - Figure 4: Distribution of cases of congenital transmission of Chagas disease by province.
- A figure of high and low vector circulation zones was unfortunately beyond the scope of this article. As discussed above, vector circulation zones were assigned directly by the local health department directors using internal data available to them by the National Chagas Program in 2019. The classification of these zones at the time did not perfectly correlate with the borders of provinces within each department. A visualization of vector circulation zones has since been published by the National Chagas Program from data in 2022, but we cannot assume these data are perfectly reflective of data available in 2019.

- In the cohort characteristics small paragraph, I would add that ...N=238 pregnant women were enrolled who were confirmed to have serological evidence of Chagas disease prior to delivery (Table 1). Something to that extent.

- We updated this sentence to the following: “From September 2020 to March 2023, 238 pregnant women with a history of Chagas disease prior to delivery were enrolled (Table 1).”

- I would also suggest, if any pictures exist from participants, which show their characteristic home structures and any pictures of triatomines inside the home. I have often found that when people are bitten, they are taking pictures of these bugs and showing them to us in the clinic. This also reinforces your findings that vector-borne transmission is still a problem and aligns with increased risk for congenital transmission.

- We did not formally collect pictures from participants of their homes or triatomines inside the home, as this was not included in the original study design and not included in our ethics review or consent process.

**Conclusions**

Reviewer #1: The conclusions are supported by the data presented and the limitations are adequately described.

The description of rates of toxoplasmosis is interesting but seems extraneous to the main study results. These could be deleted or, if not, better justification should be provided for the relevance of these results.

- As our numbers were small and not a key focus of the study, we delete our description of toxoplasmosis rates.

Reviewer #2: Each discussion is superficial such as infected babies and their increased hospitalization.

- We feel our discussion appropriately addresses a number of topics pertinent to our study, including our key findings, the importance of multiple high sensitivity testing modalities for diagnosis of congenital Chagas disease, a brief review of outcomes among congenitally infected infants, a discussion of housing characteristics associated with high vector circulation zones, differences in congenital transmission rate by vector circulation zone, a comparison of congenital transmission by parasite load, a comparison of participant characteristics by hospital region, and limitations of the study. We acknowledge that our discussion of the outcomes of infected infants is brief, but detailed infant outcomes past the initial hospitalization for birth were beyond the scope of this study.

Reviewer #3: In the discussion the authors discuss the high and low vectorial circulation zones but I can only find a reference from 2007. The results of congenital transmission align with previous studies and help confirm that there is a significant ongoing concern for congenital transmission. In my opinion, I think it is even more important to show the vectorial transmission is still abundant, likely evolving and possibly worsening. Bolivian national program for vector management has a long history of tackling T. infestans and success has been seen but studies like this are suggesting some regions have vector transmission problems. Again, I go back to a detailed map which shows your results in a way that helps strengthen your argument that vectorial transmission as it relates to congenital transmission.

- Please see our response above under “Results.”

**Editorial and Data Presentation Modifications**

Reviewer #1: Abstract. Would be helpful to include the denominator (and possibly numerator) for the proportion of congenital transmission rates by vector circulation zone.

- We updated our abstract to include both the numerator and denominator for congenital transmission rates by vector circulation zones. It now reads, “Congenital transmission rates did not significantly differ between women from low or high vector circulation zones (10.1% [9 of 139] vs. 6.5% [10 of 99], p=0.31).”

  References. The format of the article titles varies with some all in title case and others only having the first letter capitalized. The latter is generally preferred. In addition, species names, primarily Trypanosoma cruzi, are not presented in italics.
- We updated our references such that titles have only the first letter capitalized. Species names were written in italics.

  For the tables, it would be helpful to have all abbreviations defined in a footnote (e.g., SD).
- SD was written out in the description (“SD: standard deviation”).

Reviewer #2: (No Response)

Reviewer #3: (No Response)

**Summary and General Comments**

Reviewer #1: This is an interesting study that has attempted to tease out the differences in congenital Chagas disease transmission stratified by vector circulation zone. While there are several important differences in the characteristics of the women in the two zones, there does not appear to be a significant difference in risk of transmission of T. cruzi from mother-to-child in the two vector circulation zones.

This highlights the importance of PCR for testing newborns whose mothers are serologically positive for Chagas disease, the negative impact of congenital Chagas disease (based on increased rates of hospitalization although it would be helpful to have longer follow-up on these infants to determine whether they have any problems with growth or cognitive development), and the need for improved strategies for tracking mothers and their infant to ensure appropriate postpartum testing.

Reviewer #2:

The authors present recent data on mother-to-child transmission of Chagas disease in three major departments of Bolivia: Santa Cruz, Cochabamba, and Chuquisaca. The diagnostic procedures, including serology, microscopic examination, and PCR, are generally appropriate. However, some ambiguous results need further scientific evaluation. While it is crucial to prioritize the control of this transmission route, the report does not address the current low treatment coverage, which limits its potential impact.

The reviewer cannot recommend this paper for publication in *PLOS Neglected Tropical Diseases* due to the following major concerns:

1. The regional Ministry of Health’s data on natural transmission may not be fully reliable for each location.
2. There is a lack of clarity in the statistical reporting from the National Chagas Program. Especially in the different communities’ or hospitals’ situation.

- We acknowledge the reviewer’s concerns about reliability and lack of clarity in reporting of vector circulation zones from the National Chagas Program. However, we argue that there is no more reliable method of obtaining accurate and up-to-date data about vector circulation in Bolivia than conferring directly with the local health department directors, who have access to internal data from the National Chagas Program. An innovative program with almost three decades of experience, the National Chagas Program is an international leader in epidemiological surveillance and the promotion of prevention and care for a highly neglected tropical disease. We are not aware of any more reliable way of obtaining local epidemiological data in Bolivia.

3. The discussion on the correlation between parasite burden and transmission appears inaccurate.

- We are uncertain as to which part of our discussion of parasite load appears inaccurate. In our results, we state that “Parasite load was significantly lower among women who transmitted the infection compared to women who did not (33.2 ± 2.5 vs. 26.3 ± 3.5 parasites/mL, p=0.0004).” In our discussion, we report, “Our study found a significantly lower parasite load among women who transmitted *T. cruzi* infection to their infants compared to women who did not transmit the infection. This result contrasts with several past studies which have shown an association between higher parasite load and *T. cruzi* transmission risk (11, 22, 25-27). Unfortunately, parasite load measurement was limited to a very small subset of our population due to cost limitations, making these data difficult to interpret.”

4. The technical quality of microscopic exams varies significantly across regions, raising concerns about data consistency.

- We agree that micromethod has less than ideal sensitivity and specificity in part due to variation in technique. This has been previously documented and is described in our study. As a result, all infants had both micromethod and PCR testing performed at birth at one month, as well as ELISA at 9 months (for those who completed follow up). Of these 19 infants diagnosed with congenital *T. cruzi* infection, only two were diagnosed using a positive micromethod alone.

Reviewer #3: (No Response)
