# Supplementary material for: Congenital transmission of Chagas disease by vector circulation zone in Bolivia
Source: PLoS Negl Trop Dis. 2025 Oct 3;19(10):e0013591. doi: 10.1371/journal.pntd.0013591 (PMC12510653; doi:10.1371/journal.pntd.0013591)
Supplement: Attachment — Submitted filename: Response to Reviewers 7.29.25.docx [file pntd.0013591.s008.docx]

**Manuscript ID** PNTD-D-24-01292

Title: “Congenital Transmission of Chagas Disease by Vector Circulation Zone in Bolivia”

**Response to reviewer comments**

**Methods**

Reviewer #1: The authors have done a fine job of responding to my reviews and those of the other reviewers. I have no additional suggestions.

- We thank the reviewer for this comment.

Reviewer #2: I couldn't find any specific question that the authors posed in this cohort study regarding the density of the triatomine vector

- Studying the geographical density of the triatomine vector was not within the scope or goals of this study. These data are studied by the Bolivian Department of Health and can be reviewed in the National Action Plan for Entomology and Integrated Vector Management (see Reference 1).

Reviewer #3: Methods updated at the request of the 3 peer reviewers and Editorial team.

- We thank the reviewer for this comment.

Reviewer #4: Multiplex qPCR Protocol: The multiplex qPCR protocol, targeting T. cruzi satDNA and the Exogenous Internal Amplification Control (IAC), is a well-established and widely validated approach in the literature for parasite detection and quantification. It would be crucial for the authors to cite the specific reference (or references) for this widely diffused protocol to ensure methodological traceability and transparency.

- We updated this section of our methods to include three appropriate references: Duffy et al. (2009), Mayta et al. (2019), and Duffy et al. (2013).

  qPCR Reaction Description: The current description of the qPCR reaction within the Methods section requires careful review and rewriting. The phrasing "Real-time PCR was performed using 3 µl of Internal Amplification Control (IAC) added to 300 µl of the sample" it is not quite correct. It is likely that the initial sample volume for DNA extraction was 300 µL, and that 5 µL of the extracted DNA (to which the 3uL IAC was added before, at the thime of DNA purification) were then used in the qPCR reaction. The authors must precisely clarify the volumes of DNA used in the final reaction mix, as well as the stage at which the IAC was introduced (added to the sample at the beginning of extraction or to the extracted DNA before qPCR). This is vital for the comprehension and replicability of the assay.
- We updated our description of the PCR reaction in our methods according to the reviewer’s suggestions, ensuring we described the DNA volumes and the stage at which IAC was introduced. This section now reads as the following:

PCR was performed on venous samples at delivery at the Infectious Diseases Research University of the Universidad Peruana Cayetano Heredia in Lima, Peru according to established protocols (14-16). Multiplex PCR targeting *T. cruzi* satellite DNA and an exogenous Internal Amplification Control (IAC) was performed. The IAC plasmid was added at 3 µL per sample lysate prior to extraction.

Each 300 µL blood clot sample was combined with the IAC and subjected to mechanical lysis using the FastPrep®-24 Instrument (MP Biomedicals, Solon, OH, USA) at 5.5 m/s for 30 seconds in Lysing Matrix E tubes (MP Biomedicals). DNA was subsequently purified using the High Pure PCR Template Preparation Kit (Roche Diagnostics, Mannheim, Germany), following the manufacturer’s instructions. DNA was subsequently purified using the High Pure PCR Template Preparation Kit (Roche Diagnostics, Mannheim, Germany). For quantitative PCR, 5 µL of extracted DNA were used as template. Amplifications were carried out in a total reaction volume of 20 µL using the FastStart Essential DNA Probes Master (Roche) and run on the LightCycler® 96 System (Roche Molecular Systems Inc., Pleasanton, CA, USA). Data were analyzed using LightCycler® 96 software version 1.1.

Equipment and Analysis Software: For completeness and reproducibility, the authors should specify the real-time thermal cycler equipment used for the qPCR (e.g., "Applied Biosystems StepOnePlus", "Bio-Rad CFX96", etc.) and the software employed for analyzing the qPCR results (e.g., "StepOne Software v2.3", "CFX Manager Software", etc.). This information is standard and essential in articles utilizing molecular techniques.

- As above, we added the following description of the equipment and software to our methods: “Amplifications were carried out in a total reaction volume of 20 µL using the FastStart Essential DNA Probes Master (Roche) and run on the LightCycler® 96 System (Roche Molecular Systems Inc., Pleasanton, CA, USA). Data were analyzed using LightCycler® 96 software version 1.1.”

  Geographical Representation of Endemic Regions: The authors state in the Introduction section that "Bolivia is composed of nine political regions known as departments; of these, six are endemic for T. cruzi." This information is of paramount importance and relevance for contextualizing the study, especially given the manuscript's focus on the "vector circulation zone" and its impact on congenital transmission. It would be highly recommended that the authors provide a map of Bolivia highlighting the nine political regions (departments), with particular emphasis on the six regions considered endemic for T. cruzi. A clear and well-labeled map would not only reinforce the importance of the study but also help justify the experimental design and the relevance of the areas addressed.
- Per the reviewer’s suggestion, we added a new Figure 1, which shows a map of Bolivia illustrating the nine departments and highlighting those endemic for *Triatoma infestans*, the primary vector of *T. cruzi* in Bolivia.

**Results**

Reviewer #1: The authors have done a fine job of responding to my reviews and those of the other reviewers. I have no additional suggestions.

- We thank the reviewer for this comment.

Reviewer #2: The presentation of the results is descriptive, and the analysis is superficial due to the lack of a clear hypothesis. The GIS figures are difficult to interpret, and the most important entomological data are outdated and not original.

- We have revised our GIS figures according to the suggestions made by Reviewer 4. As stated above, the entomological data in our study are presented to help give the reader a frame of reference for understanding Chagas disease in Bolivia but are not intended to provide original data. They are based on the most recent updates provided by the Bolivia Department of Health from 2025.

Reviewer #3: Results were significantly improved. Figures added as requested and improve the visualization of the results.

- We thank the reviewer for this comment.

Reviewer #4: Figure 3 presents a map of participating women with Chagas disease by province, and its inclusion is valuable for visualizing the geographical distribution of the studied population. However, the legend associated with the image requires significant improvements to ensure its comprehension. Specifically in the clarification of Colors: The current legend does not elucidate the meaning of the different colors used on the map. It is essential that the legend clearly indicate what each color represents (for example, different levels of endemicity, different provinces within a department, etc.). Without this information, the reader cannot interpret the spatial distribution of the presented data. Also, the interpretation of numbers: The legend mentions a "number of women," but it does not explain what this number represents in this context. For each province (or geographical unit represented), it is crucial that the legend clearly specify what the associated number indicates (for example, the total number of participating women with Chagas in that province, the number of confirmed cases of congenital transmission, etc.). It is suggested that the authors revise the Figure 3, providing a detailed explanation of the meaning of the colors and the numbers presented on the map, making the information accessible and understandable.

- For this figure (now Figure 4), the colors on the map refer to various ranges of participating women in the study by province. To clarify this, we revised the legend with the following added label: “Range of participating women with Chagas disease by province.” For consistency’s sake, we added a similar revision to the legend in Figure 5.

  The data concerning parasitic load are of great interest and represent one of the manuscript's most valuable aspects. Despite the methodological limitations already acknowledged by the authors, the analysis and discussion of these results could be significantly elaborated to extract the maximum information from the already collected data.

  The following additional explorations are suggested:

  Comparative Analysis of Parasitic Load: It would be extremely beneficial for the authors to present a comparative graph of parasitic load between the two main groups: women who transmitted the infection versus women who did not. This visual comparison could reveal important patterns and strengthen the discussion on the relationship between maternal parasitic load and the risk of congenital transmission.

  Also, exploring the correlation between maternal and/or neonatal parasitic load and clinical outcomes in infants, such as the need for hospitalization or symptom severity, would add a crucial clinical dimension to the findings. This analysis could offer insights into pathogenicity and prognosis (Correlation with Neonatal Clinical Outcomes).
  Investigating whether infants with higher parasitic loads were concentrated in any specific geographical region could provide valuable information on local environmental or vectorial factors influencing infection or parasitic load. This connection to the "vector circulation zone" mentioned in the title would be particularly relevant.
  If the data allows, an analysis of the correlation or relationship between the mother's parasitic load and that of the infant would be highly informative. This could help understand the dynamics of vertical transmission and the impact of maternal parasitemia on the fetus.
  It is crucial that, even with the existing limitations, the authors deepen the discussion of the parasitic load data, connecting them to possible clinical and epidemiological implications. These additional analyses, if feasible with the existing data, would substantially enrich the Results section and deepen the Discussion, transforming interesting data into a more robust finding.
- Per the reviewer’s suggestion, we added Figure 6, providing a comparative graph of the parasite load of women who transmitted *T. cruzi* infection versus those who did not. We also added Supplemental Table 5, providing further characterization of the 21 total women for whom parasitic load data was available. We included data on age, vector circulation zone, department, *T. cruzi* transmission, and whether infant hospitalization at birth occurred. We added the following sentence to our Results: “Parasite load did not significantly differ by vector circulation zone or department.” However, given the very small numbers available, we feel it would be appropriate to add additional post-hoc analyses to this group. For example, only two infants of mothers with parasite load data required hospitalization; thus, it is not feasible to compare the risk of infant hospitalization by parasite load.

We also updated our discussion of parasite load to the following: “Our study found a significantly lower parasite load among women who transmitted *T. cruzi* infection to their infants compared to women who did not transmit the infection. This result contrasts with several past studies which have shown an association between higher parasite load and *T. cruzi* transmission risk (12, 24, 27-29). We did not observe a significant difference in parasite load by department or vector circulation zone. Unfortunately, parasite load measurement was limited to a very small subset of our population due to cost limitations, making these data difficult to interpret. Future studies may wish to explore differences in parasite load by geographical region and examine whether parasite load is predictive of other clinical outcomes, such as the risk of infant hospitalization.”

**Conclusions**

Reviewer #1: The authors have done a fine job of responding to my reviews and those of the other reviewers. I have no additional suggestions.

- We thank the reviewer for this comment.

Reviewer #2: The conclusions are largely inconclusive—indeed, as the authors acknowledge, the study lacked sufficient statistical power. Nevertheless, this valuable work offers a realistic perspective, and I appreciate their contribution toward strengthening public‐health strategies to prevent mother‐to‐child transmission.

- We thank the reviewer for this comment.

Reviewer #3: Conclusions are reasonable and limitations discussed.

- We thank the reviewer for this comment.

Reviewer #4: In the discussion section: The authors state: "In our study of 238 pregnant women across 11 hospitals in endemic regions of Bolivia, we identified 19 cases of T. cruzi congenital transmission to infants, resulting in a transmission rate of 8.0%. This rate is consistent with past studies; a recent meta-analysis demonstrated an average congenital transmission rate of 6.2% among women in Bolivia." It is imperative that the reference for the "recent meta-analysis" be provided.

- We thank the reviewer for noticing this error and have added the reference.

**Summary and General Comments**

Reviewer #1: As noted above, the authors have done an excellent job of responding to my reviews and those of the other reviewers. I have no additional suggestions.

- We thank the reviewer for this comment.

Reviewer #2: Chagas disease is a vector-borne illness with a prolonged, silent asymptomatic phase—both in endemic regions and beyond. Millions of chronically infected individuals remain undiagnosed and untreated. Among them, newborns are the most critically overlooked, despite being curable with benznidazole. While this study provides valuable insights into the current situation, I cannot recommend it for publication in PLOS Neglected Tropical Diseases.

- We regret this reviewer’s assessment. Our study has many strengths, including one of the largest and most geographically diverse study populations of Chagas disease in Bolivia in the last decade with participants from 11 hospitals across 3 endemic regions. We believe our publication meaningfully contributes to our understanding of Chagas disease transmission, and that we have significantly improved our manuscript through the editors’ and reviewers’ suggestions.

Reviewer #3: Author team has provided sufficient revision based off peer reviewer comments and suggestions. Thank you for your work tackling Chagas disease. I recommend accept at this time.

- We thank the reviewer for this comment.

Reviewer #4: The theme of congenital transmission of Chagas Disease in endemic zones is highly relevant, and the article contributes to the understanding of transmission dynamics in a specific Bolivian context. This is a strong point of the manuscript. The initiative to investigate this topic is commendable, as it contributes to advancing knowledge in a critical field.
Overall, the manuscript sheds light on a significant problem. However, I noted that it presents certain methodological shortcomings which, although acknowledged by the authors themselves in the Conclusion section, impact the robustness of the findings.

- We thank the reviewer for this comment. We have addressed Reviewer 4’s comments and suggestions above.
